# Stigmergy and Self-Organizing Systems in Swarm Robotics: A Systematic Review

**DOI:** 10.3390/s26134227

**Published:** 2026-07-03

**Authors:** Luigi Maciel Ribeiro, Nadia Nedjah, Luiza de Macedo Mourelle

**Affiliations:** 1 Department of Systems and Computer Engineering, State University of Rio de Janeiro, Rio de Janeiro 20550-013, Brazil; luigi@eng.uerj.br (L.M.R.); ldmm@eng.uerj.br (L.d.M.M.); 2 Department of Electronic and Telecommunications Engineering, State University of Rio de Janeiro, Rio de Janeiro 20550-013, Brazil

**Keywords:** swarm robotics, stigmergy, self-organizing, multi-agent systems, collective intelligence, swarm intelligence, bio-inspired computing, systematic review, PRISMA 2020

## Abstract

This systematic review follows the PRISMA 2020 guidelines to provide an analysis of the mechanisms of stigmergy and self-organization in swarm robotics. The purpose of this review is to conduct a bibliometric, thematic, and epistemological analysis. Journal articles addressing stigmergy, self-organization, and swarm robotics were included, whereas duplicate, irrelevant, and methodologically insufficient studies were excluded. The scientific databases searched were IEEE Xplore, ACM Digital Library, ScienceDirect, Springer Nature, MDPI, and Wiley Online Library from June 2025 to April 2026. Three reviewers independently screened studies using predefined criteria; no formal risk-of-bias assessment or meta-analysis was performed. In total, 338 scientific works were analyzed, representing a wide range of different approaches and applications in stigmergy and self-organization in swarm robotics. The results were synthesized through four complementary analytical axes. The review highlights the significance of stigmergy and self-organization principles in providing robustness, scalability, and adaptability in swarm robots, and shows the increasing popularity of hybrid solutions based on swarm optimization, distributed learning, and adaptive control. Key limitations include the fragmentation of methodologies, the lack of benchmarking, the underrepresentation of computational and physical perspectives, and challenges in multi-scale modeling. The review provides an integrated conceptual framework and identifies future research directions. This work was supported by FAPERJ (grants 201.013/2022 and 200.434/2026) and registered with the Open Science Framework.

## 1. Introduction

In recent years, progress in artificial intelligence, distributed robotics, and complexity science has promoted new computational approaches. These approaches are inspired by collective behaviors observed in nature. In this scenario, swarm robotics has emerged as a multidisciplinary research area devoted to the investigation of systems composed of multiple relatively simple robots that coordinate through local interactions to generate complex and adaptive collective behavior. As can be observed in nature through colonies of social insects, flocks of birds, and schools of fish, among others, these systems leverage mechanisms of distributed coordination, emergent robustness, and collective adaptability. Among the key processes behind this type of dynamics is stigmergy, the indirect communication mechanism based on environmental modification performed by the agents that influences the behavior of other agents and allows the emergence of collective coordination without centralized control [1,2]. The key aspect of such systems lies in the function of sensing and environmental detection. The robots in such swarms use their sensors in order to detect changes in the environment as a result of the actions performed by other robots, thus ensuring effective communication using stigmergy and adequate interpretation of information from the surrounding environment.

Stigmergy and self-organization are two concepts that, along with others like collective intelligence and distributed coordination, can be used to understand and describe the processes involved in the development of intelligent distributed systems. In swarm robotics, these mechanisms have been studied as ways to obtain interesting characteristics such as robustness, adaptability, and scalability, as can be seen in studies showing how complex collective behaviors can be created by robots following simple rules of spatial interaction, communication, and environmental perception [3,4]. In particular, sensing plays an important role in the communication between robots because it allows robots to monitor environmental markers, perform stigmergic interactions, and adapt their behavior according to the changing conditions. Aspects such as sensor noise, calibration issues, limited range of detection, and different levels of sensing capabilities are among the potential problems that may arise in such systems. In summary, stigmergy enables indirect coordination through environmental modifications, while self-organization allows local interactions to produce global adaptive behaviors; together, these mechanisms, supported by accurate sensing, form the backbone of swarm robotic systems. Therefore, distributed robotic systems relying on self-organization can perform several types of cooperative tasks, including exploration of the environment, creation of spatial patterns, and task assignment [5].

With the rise of studies in swarm robotics, several review articles have emerged to organize the theoretical underpinning of the research area. Commonly, they examine topics such as architectures and approaches used in multi-robot systems and bio-inspired methods of solving optimization and distributed decision-making problems [6,7]. Others consider collaborative architectures in heterogeneous robotic systems or new technologies and paradigms such as cyber-physical systems and distributed digital infrastructures [8,9]. Although these contributions have significantly advanced the organization of the field, many of them have only organized the literature around particular application domains or classes of algorithms, not considering stigmergy and self-organization as central topics in swarm robotics [10,11]. The diversification of research lines that investigate collective robotic systems from different angles, focused on applications or technologies, is also visible in the literature [12,13]. Thus, there is currently a scientific gap in this area in relation to the conceptual integration of stigmergy and self-organization as mechanisms in the emergence of collective behavior in distributed robotic systems, since few studies investigate their interaction systematically in this process [14].

Considering this state of the art, this paper proposes a review aiming to organize the literature and provide a systematization of stigmergic and self-organizing mechanisms used in distributed robotics. Through an integrative analysis of the literature, the study attempts to articulate and characterize the major themes in the area, such as theoretical foundations of collective intelligence, coordination architectures in multi-robot systems, and bio-inspired methods, establishing connections between biological principles, computational models, and robotic applications. Hence, this paper proposes to contribute to the construction of an integrated conceptual framework that helps to understand the scientific evolution of the area and the trends in research on swarm robotics. This study follows the PRISMA 2020 guidelines [15] for systematic reviews, adopting a transparent and reproducible protocol for literature identification, screening, eligibility assessment, and inclusion. The review was designed to ensure methodological rigor through predefined search strategies, eligibility criteria, and structured analysis procedures. In order to achieve this objective, the present work pursues the following specific goals:

1. Define and differentiate the concepts of stigmergy and self-organization in the context of distributed robotics, establishing their conceptual foundations and relationship with the principles of collective intelligence and distributed coordination.
2. Explain how indirect coordination mechanisms are applied in distributed control architectures and multi-agent systems, discussing these mechanisms in light of bio-inspired computational methods and algorithmic strategies present in the literature.
3. Identify prevailing trends, emerging applications, and paradigm shifts in swarm robotics, emphasizing the importance of hybrid solutions combining distributed optimization, adaptive learning, and physical control in collective systems. This includes leveraging macro-level bibliometric statistics such as countries, journals, and authors to map the state of the field and support the identification of trends and gaps.
4. Evaluate methodological difficulties and research gaps found in the literature, such as experimental fragmentation, lack of benchmarking, and challenges of physical-computational interactions, as well as future perspectives.

The article is divided into eight sections. First, the Introduction discusses the scientific background of this research and its objectives. Next, the section Related Works analyzes previous papers, focusing on gaps found in the literature. In the Methodology section, we discuss the procedures for constructing the bibliometric database and its sources. Results are structured in four sections. First, the section Macrostructure of Scientific Production offers a general overview of the scientific production of the studied area. Second, the section Analysis of Knowledge Base involves the structuring of the field by epistemological taxonomies. Third, the section Topic Dynamics and Emerging Topics covers temporal dynamics and trends of the development of research in the area. Fourth, the section Synthesis and Future Prospects synthesizes key results and identifies the prospective ways of research in the future. The conclusion outlines the paper by presenting its major contributions, limitations, and possible future research.

## 2. Related Works

A growing number of contributions about swarm robotics and self-organizing multi-agent systems have appeared in the literature over the last few decades, driven by innovations in distributed robotics, artificial intelligence, and complex systems. Such works investigate coordination mechanisms of robotic swarms inspired by nature, paying attention to stigmergic and self-organizing principles, as well as to decentralization, local agent interaction, and emergence of collective behaviors. Despite this growing literature, the field remains rather fragmented since studies mostly focus separately on algorithms, architectures, or application areas, while existing reviews usually do not systematically cover the links among stigmergy, self-organization, and distributed control mechanisms. Consequently, there is room for a more comprehensive review that synthesizes theoretical concepts, methodologies, and practical applications. Hence, the present review stands as one of the current works in the state-of-the-art of swarm robotics.

Collective coordination in multi-robot systems encompasses bio-inspired algorithms, distributed learning, and artificial intelligence, as well as technological and multi-agent systems infrastructure. Algorithmically, some of the prominent approaches for swarm coordination are particle swarm optimization (PSO) and ant colony optimization (ACO), used for cooperative exploration, path planning, and distributed optimization. For instance, Ref. [16] reviews variants, parametrization, and environmental properties related to PSO, while Ref. [17] compares both PSO and ACO, indicating that, despite conceptual similarities to stigmergy, the latter is mainly investigated as an optimization approach. From a larger perspective, a classification of metaheuristics is provided in [18], but without an analysis of the collective behavior emerging from them, which exposes a gap between algorithmic performance and self-organization. On the other hand, distributed learning and artificial intelligence are increasingly employed to enable adaptive coordination of agents that learn their policy during interaction with the environment and each other. Some works, like [19] on multi-agent reinforcement learning (MARL), investigate centralized vs. decentralized MARL paradigms, communication, scalability, and information sharing. In [20], the reviewed approaches are divided into reinforcement learning, evolutionary computing, swarm intelligence, and Graph Neural Networks, pointing out recent achievements in modeling collective dynamics and implicit cooperation.

In addition to algorithms and learning, the literature also stresses the role of technological components and multi-agent architecture. These constitute fundamental building blocks in realizing collective coordination in robotic swarms, as shown, e.g., in [21] where communication paradigms in multi-agent systems, including centralized, decentralized, and hybrid ones, are discussed. Similarly, Ref. [22] highlights the importance of robotic platforms, simulators, and distributed infrastructures for validating swarm intelligence concepts in realistic environments. In the context of aerial robotic swarms, Ref. [23] emphasizes the importance of trajectory coordination, energy efficiency, and real-time adaptation for autonomous UAV missions operating in dynamic and uncertain environments. Ref. [24] analyzes cybersecurity vulnerabilities and their mitigation in robotic networks, whereas Ref. [25] reviews sensing and unmanned aerial vehicle (UAV) localization in adversarial environments. Additionally, literature reviews of multi-agent system applications in complex fields indicate that multi-agent systems ensure flexibility, interoperability, and integration of digital and physical domains [26]. In addition, Ref. [27] divides robotic multi-agent cooperation into multiple layers, including distributed consensus approaches.

The combination of these related works highlights several different approaches to studying the literature on distributed collective systems, which include topics such as swarm intelligence and metaheuristic algorithms [16,17,18], distributed learning and artificial intelligence [19,20], technology infrastructure [21,22,24,25], and multi-agent systems for solving complex tasks [23,26,27]. This literature addresses the following areas: algorithmic efficiency, adaptive cooperative behavior, communication protocols, security and sensing capabilities, as well as architectures for industrial engineering applications, thus forming an interdisciplinary field which connects distributed robotics, artificial intelligence, and complex systems. The characteristics of different streams of literature reviewed are presented in Table 1, enabling the identification of converging themes, diverging methodologies, and conceptual gaps in current research on the topic.

Nevertheless, there are significant structural limitations in previous literature: almost all works discuss either algorithms, infrastructure, or architectures, but ignore potential interactions between these elements leading to collective behaviors; no analysis is provided on the explicit connection between stigmergy, self-organization, and the problem of distributed cooperation and multi-agent learning; finally, there is an abundance of work conducted on simulation platforms or application in specialized tasks, but no practical considerations of implementation for physical robots. This systematic review explicitly addresses these limitations by analyzing the current state-of-the-art. The study synthesizes conclusions strictly based on the analyzed corpus. This approach allows a comprehensive understanding of prevailing trends, gaps, and interconnections in the literature, highlighting how stigmergy and self-organization interact to support collective behavior in distributed robotic systems, and providing a solid foundation for future research.

## 3. Methodology

This systematic review was conducted in accordance with the PRISMA 2020 guidelines [15]. The review protocol was prospectively registered in the Open Science Framework (OSF) and is available at https://osf.io/wz5et (accessed on 1 June 2026). A detailed review protocol was prepared before the literature search and is publicly available in the GitHub repository (version v1.0.0) described in the Data Availability section. This section describes the methodology adopted to conduct the systematic review on stigmergy and self-organizing systems in swarm robotics, aiming to promote transparency, replicability, and consistent analysis. These methodological procedures were developed so as to properly identify, select, and analyze the pertinent literature, considering the stigmergic mechanisms and processes of self-organization in swarm robotics systems. The purpose of defining criteria and procedures is to minimize the influence of bias, guaranteeing consistency in the construction of the bibliographic corpus, as well as the necessary parameters to compare the selected works.

The proposed methodology is composed of three complementary stages covering the corpus collection, processing, and analysis. The data collection stage includes the scientific databases analyzed, the time interval considered, the Boolean query used, and the inclusion and exclusion criteria defining the corpus of analysis. The corpus processing and normalization stage explains the techniques and methodologies used for textual cleaning, normalization of affiliations, and metadata extraction for semantic and quantitative analysis. Lastly, in the analysis structure stage, the pipeline is used to analyze the articles through statistical and conceptual models, as well as through the analysis of topic evolution and new research directions.

### 3.1. Data Collection

The systematic collection of scientific articles used in this study took place in accordance with a consistent and structured approach based on data analysis and statistical control methodologies. One of the main targets pursued throughout this process was achieving methodological consistency, traceability of the procedure, and replicability of its phases. This goal was achieved through the use of reliable scientific databases. The list of databases used in the current research is listed in Table 2. A Boolean query was developed for each database that could be used to extract studies relevant to all three phenomena, namely stigmergy, self-organization, and swarm robotics (see Table 3). Eligibility criteria were developed prior to performing the search. Studies were considered eligible if:

- They were published as journal articles;
- They were written in English;
- They dealt with concepts, models, methodologies, or applications of stigmergy, self-organization, and swarm robotics; and
- They contained sufficient methodological and technical information to allow their analysis.

Studies were excluded if:

- They were not journal articles;
- They were unrelated to the topics addressed in this review;
- They were duplicate records retrieved from multiple databases; or
- They lacked the methodological information required for data extraction and synthesis.

There were no limits on the publication date to capture both historical development and recent advancements within the subject area. We acknowledge that many important contributions in swarm robotics are published in conference proceedings. However, in this review, we focused exclusively on peer-reviewed journal articles to ensure sufficient methodological detail and consistency for bibliometric and thematic analyses. This choice may underrepresent very recent or fast-evolving studies and is therefore discussed as a limitation of this systematic review.

Study selection was performed using an automation tool written in Python. It was employed for such operations as preprocessing of metadata, sorting of retrieved records, thematic classification of studies, keyword normalization, taxonomy extraction, and validation of data consistency through automation tasks and LLM-assisted analyses. Excel tables with structured data were created during the study selection procedure. Record uniqueness was provided using DOI and title-based comparisons. Following the automated processing stage, all retrieved studies underwent manual screening to assess thematic relevance and compliance with the predefined eligibility criteria. The selection process was conducted independently by three reviewers based on the analysis of titles, abstracts, keywords, and available metadata. Any disagreements regarding study eligibility were resolved through joint discussion among the reviewers until a consensus was reached. For each eligible study, standardized metadata were collected, including title, authors, abstract, journal, volume, issue, pages, keywords, citation count, and publication year. Additional information regarding authors’ institutional affiliations, cities, and countries was also recorded whenever available.

No risk-of-bias evaluation method was used as the main focus of the current review was the bibliometric and thematic analysis of scientific articles dedicated to stigmergy, self-organization, and swarm robotics, without any intention to assess their effectiveness or compare experimental results. The use of predefined inclusion criteria, independent article screening conducted by three reviewers, and a conflict resolution strategy helped avoid possible selection biases. As no meta-analysis was carried out in the course of the current study, standard effect measures like odds ratio, relative risk, or standardized mean difference could not be utilized. Instead, the synthesis was based on qualitative, bibliometric, and thematic indicators derived from the selected studies, including publication trends, citation metrics, research topics, application domains, and taxonomic classifications.

### 3.2. Corpus Processing and Normalization

The processing of the bibliographic corpus was aimed at establishing a solid and consistent knowledge base able to sustain bibliometric analysis and to support the construction of the epistemological taxonomy required in the review’s subsequent stages. First, CSV files obtained during the literature search were aggregated into one dataset, checked for integrity, and standardized for necessary fields, like the transformation of publication dates to the respective year, deletion of records with missing information, and initial institution normalization. Management of missing data was performed at the pre-processing stage. Entries without any necessary bibliographic data (title, author(s), year of publication, or abstract) were removed from the final dataset. Whenever possible, missing affiliation data were harmonized using the information on affiliated institutions. Date information collected from various databases was transformed into a standard year format. In turn, such data standardization provided an opportunity for consistent comparison and the semantic/statistical vectors necessary for building the mentioned epistemological taxonomy and evolution of topics in the next stage.

To find the geographical locations of the authors, a pipeline for country extraction was created on the basis of institutional affiliation. Such a procedure included Unicode normalization, cleaning of common words, and mapping of institutions to the ISO-3166 standard [28]. It was required to ensure the geographical consistency and enable reliable analysis. Keywords of each article were subjected to lexical and semantic normalization, i.e., transformation to lowercase, removing accent marks and punctuation, normalization of delimiters, and semantic classification to avoid redundancies and terminological inconsistencies. The frequency of terms globally was computed to establish those terms representing the corpus most accurately and allow emerging topics to be defined on the basis of quantitative/qualitative indicators. Thus, the thematic analysis will take into account the scientific relevance of each term and ensure the semantic consistency of the whole dataset. For performing all bibliometric analysis, thematic classification, and other data processing operations, custom-made Python scripts were used. The structured datasets were stored in MS Excel files, and all statistical analysis and visualization were carried out using Python. As a consequence of this approach, a structured analytical database was created, containing the following elements for every article:

- bibliographic metadata (validated);
- normalized country of the first author;
- publication year;
- normalized keywords set;

### 3.3. Study Selection

**Figure 1:**
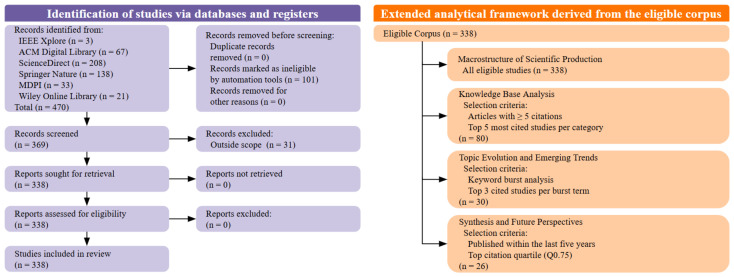
PRISMA 2020-based study selection process and extended analytical framework adopted in this review. The left side illustrates the identification, screening, eligibility assessment, and inclusion stages, resulting in an eligible corpus of 338 studies. The right side presents the analytical subsets derived from the eligible corpus for the four complementary axes of analysis: macrostructure of scientific production, knowledge base analysis, topic evolution and emerging trends, and synthesis and future perspectives.

The study selection process followed the PRISMA 2020 guidelines. The complete identification, screening, eligibility, and inclusion process is illustrated in the PRISMA flow diagram presented in Figure 1. Initially, records retrieved from all scientific databases were aggregated into a single corpus, denoted as $D_{0}$:

(1) $$D_{0}=D_{\mathrm{IEEE}}\cup D_{\mathrm{ACM}}\cup D_{\mathrm{ScienceDirect}}\cup D_{\mathrm{Springer}}\cup D_{\mathrm{MDPI}}\cup D_{\mathrm{Wiley}}$$

The resulting corpus contained 470 records. After duplicate removal and application of the eligibility criteria, the eligible corpus ($D_{E}$) was obtained:

(2) $$D_{E}=\left\{d_{i}\in D_{0}|d_{i}\;\mathrm{satisfies}\;\mathrm{all}\;\mathrm{inclusion}\;\mathrm{criteria}\right\}$$

The final eligible corpus comprised 338 studies. Table 4 summarizes the study selection process. While the traditional PRISMA 2020 ends with the selection of a single set of studies eligible for the research question, this literature review introduces several other steps in the methodology in order to meet different aims of the analysis. Following the identification, screening, eligibility assessment, and inclusion criteria described by PRISMA, the corpus, denoted as $D_{E}$, was obtained has been analyzed through four different angles aimed at the detailed characterization of the field.

Four analytical axes, their goals, and respective subsets of studies analyzed are briefly presented in Table 5. The first axis is called the macrostructure of scientific production. It involves the analysis of the overall structure of the scientific production based on publication features, geography, citation indicators, and collaboration networks. The second axis, called knowledge base analysis, aims at exploring the conceptual basis of the field by means of its organization in a thematic taxonomy hierarchy and the measurement of thematic importance based on citation numbers. The third analytical axis, called topic evolution and emerging trends, is dedicated to the exploration of the temporal structure of the scientific literature via the detection of the evolution of concepts and emerging trends. The fourth axis, called synthesis and future perspectives, seeks to identify the key issues addressed in recent literature and the potential areas for future research.

In line with the previously described objectives, dedicated sets of articles have been selected from among the eligible ones according to PRISMA requirements for traceability. In particular, in regard to the Macrostructure of Scientific Production analysis, all eligible studies (n=338) are utilized. This analytical layer includes several bibliometric measures characterizing the scientific production in terms of geography, dynamics, impact, and co-authorship structures. Specifically, publication trends by country and author names, yearly publications dynamics, citation trends, and international collaboration patterns have been assessed to identify the key countries, authors, journals, and collaborative structures in the field. This layer gives an overall picture of the research field and serves as a platform for further conceptual and thematic analyses.

With regard to the Knowledge Base Analysis axis, articles are subject to systematic taxonomization according to thematic synthesis and content-based criteria. The taxonomic analysis is based on thematic analysis and thematic synthesis principles [29,30]. As a preparatory stage before conducting thematic classification, the knowledge base has been formed by joining titles, abstracts, and keywords of the articles together into one textual piece for each article. The knowledge base has been cleaned of duplicates and irrelevant terms. Next, the term importance has been estimated by textual and bibliometric measures such as publication frequency, citation impact, and thematic distribution in the literature. Thus, the terms describing the most important features of the knowledge domain could be identified.

The emerging recurrent themes are then revealed, taking into account epistemological, methodological, architectural, and application-related perspectives. Discovered themes were categorized and refined into a hierarchical taxonomy consisting of thematic macrocategories and subcategories using qualitative content analysis principles. To enhance conceptual consistency and avoid classification bias, a taxonomic structure was obtained by iteratively extracting candidate topics from article groups, removing duplicate concepts, and hierarchically structuring these concepts into thematic centers and subcenters. Afterward, each article was assigned to a thematic category that represents the main contribution of the study, thus allowing analysis of the distribution of studies in the conceptual space and identifying the dominant research lines.

In order to analyze each category in depth, a subgroup of highly cited articles in each thematic category is selected based on citation counts. Only articles with no fewer than five citations are taken into account initially, indicating the minimum required impact and recognition. Then, studies are sorted in descending order according to citation counts within each thematic category, and the five most highly cited articles in each thematic category are selected as representatives. This leads to the identification of the most cited contributions in each thematic category and a subset of 80 articles.

As far as the topic evolution and emerging trends axis is concerned, yearly keyword frequencies have been counted after normalization and aggregation. Further, year-over-year growth rates have been computed for each keyword, allowing fast-growing topics to be identified. At the next step, burst detection methods have been used to determine the keyword with the maximum positive growth rate per year without duplication of terms in the output. This has led to obtaining the timeline of topic emergence and evolution. Then, the three most highly cited articles published in the year of interest with the selected keyword have been chosen for each emergent term. By doing this, the relationship between emergent topics and the corresponding contributions is directly established, yielding the subset of 30 articles. Finally, for the Synthesis and Future Perspectives axis, only papers published in the last five years are selected. From the selected papers, only those included in the upper citation quartile ($Q_{0.75}$) are chosen, leading to a subset of 26 papers.

The results of the review are presented using complementary tabular and graphical representations. The study selection process is summarized through the PRISMA 2020 flow diagram, Figure 1, while descriptive statistics are reported in summary tables. Bibliometric indicators, temporal publication trends, geographical distributions, collaboration networks, thematic taxonomies, and topic evolution are presented through tables and graphical visualizations generated using custom Python scripts. These visual representations were adopted to facilitate comparison across studies and to support the synthesis of the identified evidence.

## 4. Macrostructure of Scientific Production

The general overview analysis allows us to obtain a macrostructural view of the literature onstigmergy and self-organizing systems in swarm robotics, which is useful to understand the development pattern of academic production, the distribution of academic production, and even the structuring of scientific community networks. The dataset under study was obtained through a systematic literature review performed between July 2025 and April 2026, yielding the consolidation of a corpus of 338 papers published in scientific journals. The dataset generated and analyzed during this study, together with the review protocol, is publicly available in the GitHub repository (https://github.com/LuigiRibeiro/SSOS-SR-Dataset, accessed on 1 June 2026). In general, the examination of the essential aspects regarding the production of articles in terms of quantity and structure helps to verify whether the field is at a mature phase, considering development periods of emergence of concepts, methodological development, and institutional consolidation, especially in light of advances in the area of bio-inspired algorithms, multi-robot systems, and collective intelligence. Beyond the quantification of the production in terms of the bibliometrics, the findings are structured so that the macro-structural configuration of the field can be seen, pointing out convergences of research topics, academic communities, and methods. In this context, the dynamics of scientific production growth are explored in terms of article volume and distribution of the production in relation to authors, countries, and institutions, in addition to the identification of main hubs of research and key players. Thus, one can explore the formation process of a community of scientific research in the field of swarm robotics.

**Figure 2:**
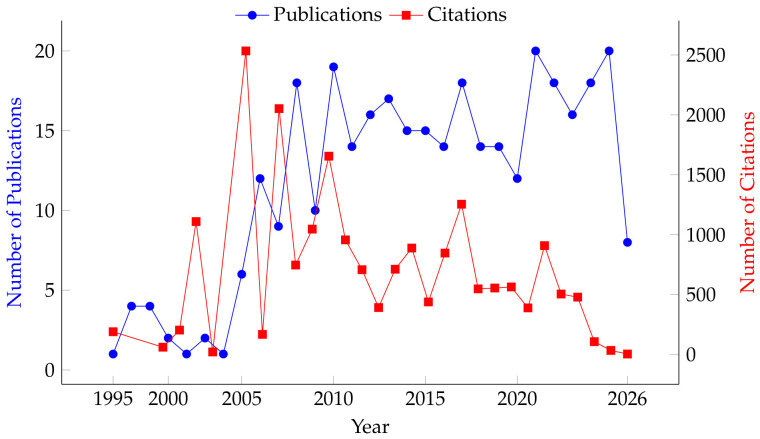
Temporal evolution of publications (blue) and citations (red) within the analyzed corpus. Sparse initial growth is observed during the 1990s, followed by consistent expansion between 2005 and 2013, stabilization from 2010 onward, and renewed interest beginning in 2021. Citation peaks in 2000, 2003, 2005, and 2008 reflect highly influential foundational works, while recent years show lower counts due to shorter citation accumulation windows.

In Figure 2, one may observe the time series for publications and citations in the analyzed corpus composed of 338 scientific papers. It is possible to identify an initial sporadic growth in the 1990s and a constant increase in the number of papers between 2005 and 2013, in which the number of articles published each year is consistently above ten publications per year. After 2010, the annual production stabilized between twelve and twenty publications, characterizing the maturation process of the scientific field. From 2021 onward, one observes growth in scientific production, reaching peaks in 2021 and 2025. As for the scientific impact, measured here by citations, one notes significant peaks of citations in 2000, 2003, 2005, and 2008, associated with highly cited foundational papers. Afterwards, there is a constant pattern of increase in the number of citations, while the most recent years present fewer citations due to their shorter citation window.

The geographical analysis of scientific production allows for identifying the main research centers and the consolidation process of the latter within the framework of international scientific collaboration. An integrated assessment of publication volume, academic impact, and international collaborations by country, as seen in Table 6, allows detecting specific structures of the global scientific field. Italy leads in the number of publications (43), followed by China (34), the United States (29), the United Kingdom (24), and France (23). Thus, there is an evident concentration of research efforts in hubs located in Europe and North America, although there are also countries from Asia that actively engage in this field of scientific research. Historically leading countries (the United States, Germany, the United Kingdom, etc.) remain consistently relevant, whereas emerging research centers (China, India, Spain, and Brazil) show a steadily increasing trend of participation within the global scientific corpus.

The analysis of the average impact of publications shows that the impact of research is not linearly proportional to the number of papers published. Belgium and Spain produce relatively few papers (16 and 13, respectively) but have a relatively high average citation rate (109.38 and 114.15). Therefore, we can say that these papers represent highly specialized publications with a significant academic impact, which are essential to the conceptual development of the topic. At the same time, Italy and China demonstrate a steady balance between their moderate publication volume and impact. The analysis of medians and maximum citation rate per paper gives us another indicator of internal diversity in academic influence. For example, the peak of academic influence for the United States is estimated at 2481, which means that there are papers with extremely high citation rates that are essential for the concept of the field. However, the median for the same country equals 21, which shows that most of the publications achieve a decent impact. Such a pattern is typical for the entire field.

Analysis of international collaborations shows the structure of global scientific networks in the field under investigation. Italy, France, the United States, China, and the United Kingdom are among the main hubs, having multiple strategic contacts that allow them to disseminate knowledge and integrate other scientific ecosystems. The role of countries having a lower volume of publication but showing a higher average citation rate is associated with the conceptual development of the field under investigation, whereas hubs are involved in the creation of the global scientific network. In conclusion, one may note that the scientific influence of a country is determined not only by publication volume but also by the potential for international cooperation.

Supplementary to the previous analyses, the examination of editorial concentration and productivity of key authors offers important information concerning dynamics and trends in knowledge dissemination in the field. Despite the presence of several notable exceptions, such as *Swarm Intelligence* (18 papers), *Robotics and Autonomous Systems* (16 papers), and *Engineering Applications of Artificial Intelligence* (12 papers), scientific production can be said to be rather distributed across various specialized journals (Table 7), reflecting the multidisciplinary nature of the topic. As a consequence, despite the presence of key journals for orientation, one should note that there is no significant editorial concentration typical of fields that are experiencing rapid evolution in terms of the emergence of novel applications, methods, and topics.

The same conclusion holds true for the analysis of first-author productivity in the field: there is no clear dominance of a particular author among researchers. Among the 22 authors publishing at least two papers as a first author, the leading author is Paul Valckenaers, publishing four papers [45,46,47,48], followed by Frederick Ducatelle with three publications [49,50,51], and the rest having only two papers each. The lack of dominance among authors confirms the highly collaborative nature of the field and the plurality of methodological approaches employed by different authors and research groups.

## 5. Knowledge Base Analysis

The analysis of the knowledge base has been carried out to identify the epistemological structure through which the field of stigmergy and self-organizing systems in swarm robotics can be understood in terms of its theoretical structure. Rather than using a purely numerical assessment of the literature, the analysis of the knowledge base relied on bibliometric and thematic classification strategies, producing an epistemological taxonomy of the corpus analyzed, showing aspects of thematic concentration, conceptual recurrence, and organization of scientific knowledge.

The epistemological taxonomy produced through this study organizes the literature reviewed in four macro-centers (foundations, methods, architectures, and applications) corresponding to distinct abstraction levels and degrees of scientific maturity. As a result, it becomes possible to identify an interesting epistemological flow: the foundations of theories explain the behavior of collective phenomena; computational methods formalize such phenomena; architectures allow for their implementation; and applications take advantage of this in practice. Regarding their distribution quantitatively, there is a strong predominance of the methods dimension (126 papers), followed by architectures (76), applications (71), and foundations (65).

**Figure 3:**
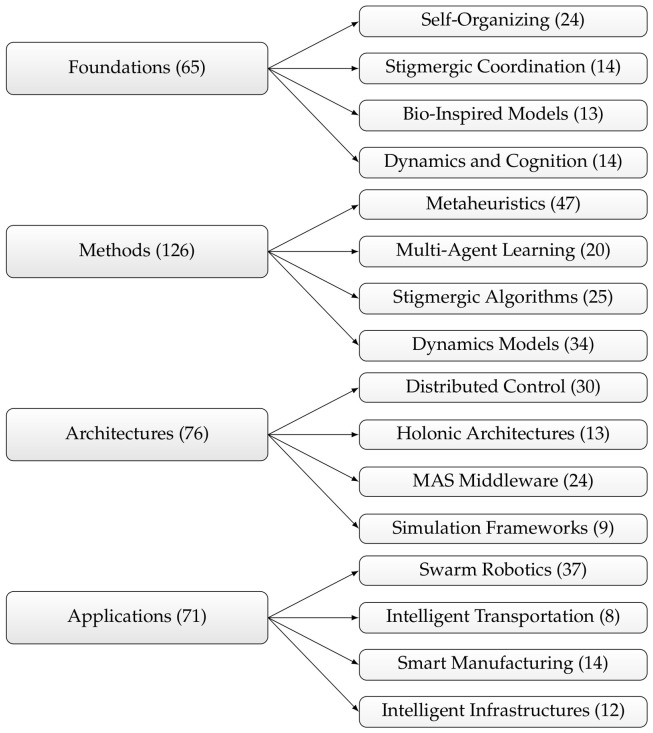
Knowledge-base taxonomy organized into macro-centers (**left**) and their respective subcenters (**right**), where the values in parentheses indicate the number of articles associated with each category.

The analysis of the subcenters, on the other hand, allows one to perceive some characteristics in the way the organization of scientific knowledge occurs. Within the Foundations macro-center, one may highlight studies on self-organization and emergence, studies regarding stigmergic coordination, and research on bio-inspired systems. Within the Methods macro-center, there is a high concentration of studies in bio-inspired metaheuristics and in the modeling of collective dynamics. Regarding Architectures, distributed control solutions and multi-agent middleware are prevalent. With respect to the Applications, a predominance of swarm robotics can be noted, indicating that this area is indeed where the results are validated. The resulting epistemological taxonomy and its hierarchical organization into macro-centers and subcenters are illustrated in Figure 3.

### 5.1. Foundations

In contrast with the other dimensions of the classification taxonomy, the Foundations macro-center includes theoretical frameworks describing how collective behaviors arise out of local interactions. Therefore, in terms of the nature of the concepts involved, Foundations does not include any notion of computational implementation or applications but focuses on the formulation of the basic principles of collective dynamics. Particularly, within this macro-center one can find theories of emergence, self-organization, collective cognition, as well as models inspired by biological systems, thus laying down the theoretical basis for the development of methods, architectures and applications.

From a structural point of view, the foundations macro-center comprises four distinct subcenters encompassing different theoretical views of collective systems. The Self-Organizing and Emergence in Collective Systems subcenter deals with the problem of studying the mechanisms of emergence of coherent patterns through the interaction among local elements without a centralized control center. Foundations of this research line were set up by [52], who outlined terminology, problems related to the design and study of collective agents, and principles for the behavior control of distributed systems. One of the most relevant issues in this research area is the distinction between top-down and bottom-up strategies. While the former presupposes starting from a global specification and distributing the control through communications, in the latter the global behavior is produced in consequence of the interaction between agents and the environment, as explained by [53]. Regarding adaptive systems, Ref. [54] introduced the idea of controlled self-organization by proposing a typology and a measure of autonomy based on internal and external control. A formal relationship between self-organization and emergence was formulated by [14] through the introduction of discrete-event systems, whereby the stigmergic systems are a specific case of complex adaptive systems. More generally, Ref. [55] presented a comprehensive review on self-organization modeling in MAS with applications to distributed networks and complex systems. In summary, studies included in this subcenter deal with topics like collective dynamics, adaptation, and emergent pattern formation and, therefore, focus on the principles of emergence.

The stigmergic coordination and indirect communication subcenter provides a theoretical perspective based on the idea of environment-mediated coordination through interactions where agents change the environment indirectly by means of signals, for example, pheromone or digitally encoded signals. Stigmergy was formally defined as an environment-mediated stimulus response process, whose foundations were developed by [56] with the aim to propose a conceptual model and standardize the meaning of stigmergy, showing that the process applies outside biology. The cognitive aspects of stigmergic interaction were studied by [57], proving that stigmergy operates at the level of many systems, such as neural networks, social systems, and is responsible for the emergence of collective cognition. From the sociotechnical point of view, Ref. [58] demonstrated that the emergence of complex systems occurs thanks to the decentralization of contributions enabled by technology in an environment mediated by stigmergy. Complementary, Ref. [59] extended the understanding of stigmergy by making connections with other coordination mechanisms and highlighting its cognitive side. In practical terms, Ref. [60] showed that in multi-robot systems, the environment can serve as a mediation channel for implicit communication, allowing coordination in the absence of explicit messages. In conclusion, studies included in this subcenter deal with the principle of distributed coordination characterized by scalability and a lack of a centralized controller.

The third subcenter included in the macro-center, bio-inspired models of collective behavior, comprises conceptual models drawn from biological systems, such as social insect colonies, chemotaxis, and natural cooperation mechanisms. The foundations of this subcenter were provided by [36] by analyzing models inspired by ants’ behavior and highlighting the role of stigmergy in distributed communication, able to explain collective self-organization in simple systems. Complementary, Ref. [35] investigated the biological fundamentals of swarm intelligence, revealing that interactions, bifurcation, and individual adaptability lead to the emergence of collective behavior and flexibility. Transposition to artificial systems of such principles was proposed by [61], who proved that local parameter adaptation in robots is enough to produce emergent task allocation without representing individuals’ characteristics. Systematization of bio-inspired mechanisms was proposed by [38], who designed a bio-inspired modular pattern repository for supporting engineering of self-organizing systems. More generally, Ref. [62] proposed the concept of synthetic collective intelligence, expanding principles discovered in biology to artificial systems or even modified biological systems capable of collective interactions and adaptation. Overall, these models form a substantial part of the conceptual base to draw out natural phenomena’ robustness, adaptability, and self-organization.

Finally, the dynamics and cognition in complex adaptive systems subcenter moves toward a system-oriented approach that comprises concepts such as multiscale dynamics, information propagation, and collective cognition. The notion of intelligent behavior in distributed systems was generalized by [63], who formulated the concept of physical intelligence, according to which intelligence results from the interaction between computational processes and the physical/material properties of the agent. Resilience of these systems was examined by [64] by considering self-healing mechanisms and other self-* properties (e.g., self-configuration, self-adaptation, self-optimization, and self-protection) at multiple levels. In the realm of a distributed network, Ref. [65] proved that distributed systems that work on local information and are based on feedback and adaptation techniques can be scalable and resilient, as in the case of MANETs and P2P systems. As to the aspect of collective cognition, Ref. [66] interpreted institutional arrangements as collective coordination mechanisms arising as a consequence of interactions between agents and Ref. [67] considered the Web as a complex adaptive system where the distributed organization of information emerges from social and spatial interactions among agents. In summary, studies comprising this subcenter prove that distributed systems are capable of adapting themselves to changing conditions and organizing the process of information processing, leading to emergent collective cognition.

### 5.2. Methods

Methods macro-center includes the collection of techniques that allow implementing the theoretical foundations of self-organization and collective intelligence into multi-agent systems and swarm robotics. This dimension represents algorithmic implementation knowledge about the theoretical foundations of self-organization and collective intelligence. It implies transformation of conceptual frameworks into computational procedures that can implement distributed coordination, collective optimization, and adaptation. As the most populated macro-center in the analyzed corpus (126 publications), it shows a pronounced tendency of research within this field towards developing computational methods and algorithms.

This macro-center consists of four subcenters reflecting different methodological paradigms. Bio-Inspired Metaheuristics and Swarm Optimization subcenter contains algorithms inspired by biological processes designed to perform exploration and optimization of a complex search space. One of the basic paradigms implemented within this subcenter—ant colony optimization—was described in detail by [37]. They systematized the biological foundation, algorithmic formulation, and applications of ant colony optimization in problem domains like routing and load balancing. Extension of ant colony optimization into the continuous problem domain has been carried out by [68]. Their approach involved using the Continuous Interacting Ant Colony algorithms that included multiple communication channels, allowing modeling emergent phenomena. Ref. [69] considered applications of bio-inspired algorithms in data mining, differentiating biomimetic algorithms and those optimized for solving specific tasks. Ref. [70] showed the applicability of ant colony algorithms in solving classical combinatorial scheduling problems by demonstrating superior performance obtained through hybridized algorithms. Finally, Ref. [71] tested the combination of ant colony optimization with particle swarm optimization in complex problems (e.g., the Traveling Salesman Problem), showing notable performance improvement. Thus, consisting of 47 works, this subcenter includes a dominant methodological stream based on heuristic strategies utilizing natural adaptation, indirect cooperation, and selection processes, but not relying on probabilistic or deep learning approaches.

Reinforcement learning and multi-agent learning subcenter involves algorithms that rely on an adaptive process where agents adjust their strategies through interactions with the environment and each other. Its foundations have been systematized by [72]. They classified reinforcement learning into two categories: team learning and concurrent learning, discussing associated issues of scaling and task decomposition. Some of the practical applications include reinforcement learning in industrial environments. In particular, Ref. [73] applied Q-learning for scheduling in manufacturing systems to enable adaptive scheduling decisions, whereas Ref. [74] developed a multi-site scheduling model involving a reactive learning strategy with competitive results. Concerning decentralized reinforcement learning, Ref. [75] offered superior solutions to complex optimization problems through distributed reinforcement learning, and Ref. [76] managed to achieve structural adaptation of agent network through integration of multi-agent learning with trust-based self-organization. These results demonstrate growing tendencies toward a combination of artificial intelligence and self-organization techniques to develop the capabilities of agents for coordination through experience-based learning.

The stigmergic algorithms and distributed coordination subcenter includes algorithms relying on interaction with the environment as an indirect means of communication among agents. With regard to path formation in robot swarms, Ref. [77] demonstrated the emergence of global patterns resulting from chains of robotic agents and distributed vector fields. Task allocation problems were successfully solved by [78] who proposed a Vacancy Chain Scheduling model inspired by natural processes, showing efficiency even when dealing with highly complicated problems. An innovative approach to path planning in robot swarms is presented in [1]. They suggested the creation of traces by movements that function as collective memory to analyze agent behaviors in practice. Another technique is based on the trophallaxis-inspired model that allows collective regulation only via interactions in a complex system adapted to changing conditions, according to [79]. Finally, Ref. [80] used chemotaxis-inspired pheromones to achieve efficient coverage in search operations. This is done through gradient-based movement combined with Lévy flights to improve performance.

Finally, the computational models of collective dynamics subcenter covers various models that help simulate, analyze, and generate emergent patterns in multi-agent systems. Ref. [40] discussed various techniques that allow accomplishing the stated purpose (such as cellular automata, multi-agent systems, and swarm intelligence). Modeling of swarms in continuous space was provided by [81] who used methods from statistical physics to represent spatial dynamics depending on agent behaviors. By contrast, Ref. [82] showed that collective phenomena like aggregation can be modeled simply by using stochastic events rather than detailed models of agent movements. Ref. [83] underlined the necessity to have a systematic model construction process and proposed a methodology to do so. Ref. [84] developed the probability collectives framework based on simultaneous optimization of local utilities of agents with convergence to the global objective in combinatorial optimization tasks.

### 5.3. Architectures

The ‘architectures’ macro-center represents a dimension dealing with enabling the practical deployment of computational methods in distributed multi-agent systems and swarm robotics. While the previous two macro-centers focused on theoretical concepts or algorithms, this macro-center concentrates on practical issues related to software, hardware, and middleware infrastructure used to coordinate, support communication in, and implement multi-agent systems. Thus, this macro-center reflects the increasing importance of issues such as scalability, interoperability, and system integration, which are characteristic of the transition from theoretical to applied multi-agent system development.

This macro-center includes four subcenters covering various aspects of distributed agent system development. The distributed control architectures and swarm robotics subcenter deals with approaches used to enable decentralized coordination in multi-robot systems. In particular, Ref. [85] provides a general view on the problem by introducing a taxonomy based on multiple dimensions like communication, planning, and decision making, and explaining how different approaches influence robot performance in tasks like exploration and cooperative transportation. The modular nature of multi-robot systems was investigated by [86] in the form of the MITE framework that considers robotic systems in terms of modules, information, tasks, and environment. Moreover, Ref. [87] showed the efficiency of evolutionary controllers in creating robust behaviors in physical robots. With regard to cooperative navigation, Ref. [49] proposed a delay-tolerant communication approach that enables emergent formation of effective path-discovery structures. Finally, Ref. [88] discussed the trend from traditional monolithic architectures to hybrid multi-component systems and their role in coordinating robots working in dynamic environments. Overall, this subcenter comprises the dominant current in the field, being characterized by swarm robotics as a validation environment for multi-agent system methods and algorithms.

Another subcenter related to industry-scale distributed agent systems, the holonic architectures and cyber-physical systems, focuses on the use of holonic systems and cyber-physical systems in enabling smart manufacturing and implementing Industry 4.0 ideas. Ref. [89] provided a detailed analysis of the basic components of these systems as integration of MAS, SOA, and self-organization. Within the context of planning and control problems, Ref. [90] found that a combination of centralized and distributed approaches to control systems is necessary to prevent suboptimal behavior and achieve high-performance logistics. The significance of the holonic paradigm for advanced industrial environments was examined in [91], where authors discussed its contribution to the main enabling technologies of Industry 4.0 and described several research directions to be addressed in the future. A practical solution to the problem was provided by [92] in the form of an implemented agent-based manufacturing execution system organized as cooperative holons and tested in an industrial environment. Additionally, Ref. [93] extended this concept by using the paradigm in designing intelligent cyber-physical systems for distributed space operations. Overall, these works demonstrate the maturity of the industrial applications of multi-agent systems, with interoperability, adaptability, and reliability as key components of the solutions.

The middleware and multi-agent coordination infrastructures subcenter is dedicated to the infrastructure used to implement distributed multi-agent systems. One of the most valuable theoretical contributions related to this topic belongs to [94], who introduced an interesting definition of environment as a first-class entity providing the infrastructure for interaction and indirect coordination between agents. This idea was then developed by [31] in the form of the agents & artifacts metamodel, in which artifacts represent reactive entities enabling interactions and thus shaping the execution environment. Coordination via tuple spaces in distributed MAS was studied by [95], who proposed the corresponding formal model of interaction. In addition to this, Ref. [96] considered bio-inspired coordination models as potential middleware solutions to problems specific to pervasive computing environments. Finally, Ref. [97] demonstrated practical implementation of such infrastructure by proposing a novel asynchronous communication-based architecture for distributed swarm algorithms implementation. All these works highlight the importance of the infrastructure layer as an enabler of the separation of application code from technical aspects of multi-agent systems implementation.

Finally, the experimental platforms and simulation frameworks subcenter covers various aspects of validation and experimentation of multi-agent systems. To begin with, a comprehensive review on the state-of-the-art of these platforms was published in [32]. It provides an overview of different approaches to modeling and simulation and highlights several popular modeling methods, simulation tools, and swarm robotics platforms used in solving problems such as navigation and searching. Further discussion on the combination of modeling and simulation was made by [98], who proposed applying the DEVS formalism for addressing emergent properties of complex systems. Multi-level modeling and simulation was studied by [99] in the form of developing a multi-level simulator able to switch between various abstraction levels to achieve the best trade-off between modeling and computational efficiency. In the experimental field, Ref. [100] proposed using Kilogrid as a physical platform for performing experiments with Kilobots. At the same time, Ref. [101] introduced a methodology for building agent-based simulators in non-deterministic environments, providing gains in efficiency and scalability. Despite being the smallest in terms of publication volume, this subcenter plays an extremely significant role since it provides the necessary experimental framework for validating theoretical and algorithmic work on multi-agent systems.

### 5.4. Applications

Applications constitute the macro-center representing the application of theoretical principles, computational methods, and architectures towards solving real-world problems. This category involves the diverse areas in which multi-agent systems and swarm-inspired algorithms find application, thus indicating the maturity of the discipline and its technological transfer potential. As the name implies, this macro-center demonstrates the application orientation, although the volume of publications is relatively smaller compared to the Methods dimension, implying the dominance of theoretical research within the field.

The internal structure of this macro-center is formed by four subcenters covering the main application domains. Firstly, the Swarm Robotics and Cooperative Autonomous Systems subcenter is highly represented, consisting of 37 papers, and thus constitutes the major field for applying distributed coordination mechanisms. The basis of decentralized cooperative transportation was introduced by [39], demonstrating how robots can resolve tasks through interacting locally without communication. The emergence of collective decision-making was studied by [102], showing how swarms could choose optimal solutions relying purely on physical interactions. Critical scenario applications were reviewed by [103], discussing multi-robot systems used for search and rescue operations in terms of challenges and potential advantages. Resource-limited conditions for operating swarms were explored by [104], showing emergence of coordinated behavior through local communication. Finally, Ref. [105] used drone swarms for suppressing wildfires, leveraging the robustness and scalability of physical and self-organizing mechanisms. Taken together, this body of literature proves the ability of decentralized systems to adaptively perform complex tasks like transportation, exploration, and emergency assistance.

Intelligent transportation systems and vehicular networks subcenter represents the domain where swarm-based algorithms and multi-agent coordination mechanisms are applied to vehicular communication networks and traffic control. An exhaustive review covering this topic was done by [106], organizing vehicular routing tasks and reviewing bio-inspired metaheuristics. The use of the Ant Colony Optimization algorithm for addressing the routing problem was studied by [107], presenting the EHACORP protocol. Ref. [108] leveraged ant-based behavior to optimize urban traffic flows by selecting less crowded routes dynamically. Integration of swarm intelligence in vehicular networks was examined by [109], showing increased efficiency of communications with decreased latency. Digital pheromone models for predicting vehicular flows were suggested by [110], employing local interaction of agents for achieving the goal. Despite being an emerging area of research, this domain holds significant potential due to the growing complexity of transportation infrastructure and the effectiveness of decentralized approaches in dynamic environments.

The smart manufacturing and distributed industrial systems subcenter represents the area in which applications involve industrial planning, control, and automation using multi-agent systems. The integration of ant colony intelligence into decentralized coordination was studied by [111], introducing the dynamic scheduling model for managing allocation and sequencing. Adaptation and reconfiguration of industrial systems using bio-inspired methods were discussed by [112], demonstrating how such principles could increase flexibility and resilience. Agent-based models were applied for autonomous manufacturing systems by [113], focusing on modeling emergent behavior in distributed systems. Scheduling problems under uncertain conditions were considered by [114], employing a holonic approach along with hybrid methods to optimize performance. In addition, Ref. [115] used a potential-field-based approach for developing dynamic control in flexible manufacturing systems, allowing for adaptive resource selection. Overall, these studies prove the maturity of multi-agent systems application in manufacturing, emphasizing its ability to support adaptability and flexibility.

Finally, the intelligent infrastructures and monitoring systems subcenter represents applications of multi-agent systems and swarm-based algorithms in distributed infrastructures, including environmental, smart grids, and city applications. The issue of distributed data gathering in vehicular networks was approached by [116], proposing a strategy for directing agents to the regions based on chemotaxis and Lévy flight mechanisms. Ad-hoc UAV networking was researched by [117], using a pheromone-based model for optimizing navigation and prediction of energy consumption. In the environmental domain, Ref. [118] suggested an agent-based model for simulation of the ecological process by combining swarm intelligence with geospatial data. The development of a distributed framework for knowledge discovery in natural disasters was studied by [119], employing bio-inspired and geospatial analytics. Finally, recent works such as [120] focused on the management of smart electrical grids, highlighting the benefits of decentralization compared to centralized control. Overall, this body of literature demonstrates the applicability of multi-agent systems for monitoring and management of critical infrastructures.

## 6. Topic Evolution and Emerging Trends

Evolutionary analysis is a valuable tool for understanding the development of the field of swarm robotics and self-organization. The results obtained using the methodology will allow us to understand not only how production in this area grows but also how the field itself develops and what changes occur. Since it is an interdisciplinary area in which artificial intelligence, complex systems, and multi-agent systems engineering interact, it becomes possible to trace how the paradigms of the field change, new approaches appear, and the focus changes. Thus, evolutionary analysis provides a macrostructural picture of the field of swarm robotics.

Keywords’ frequency metrics, co-occurrence networks, and burst detections are used for identifying central concepts, semantic relations, and periods of increased scientific activity. On the basis of such analysis, the discussion about the evolution of the topic will be organized through the following three dimensions. First, it will cover the identification of recurring concepts and semantic links between them. Second, the discovery of emerging topics. Third, the temporal analysis of research streams. This approach will help to understand how the discussed concepts, such as stigmergy, self-organization, and distributed coordination, develop, interact, and change.

**Figure 4:**
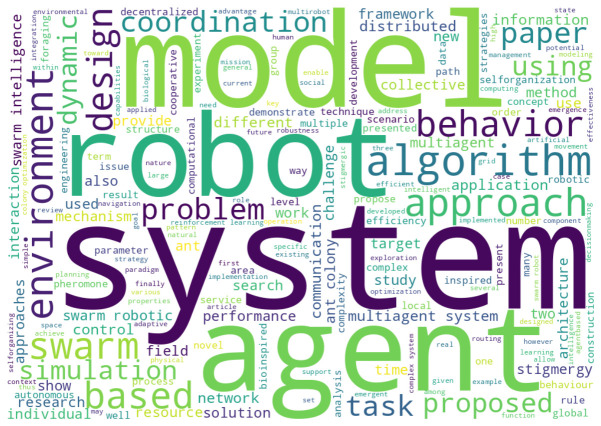
TF-IDF-based word cloud highlighting the relative relevance of terms within the analyzed corpus and illustrating the main conceptual axes of the field, including swarm robotics, multi-agent systems, computational methods, and distributed coordination mechanisms.

Global keyword frequency, together with TF-IDF weights, shows that the knowledge base is based on a strong concept-centered structure of collective intelligence, distributed coordination, and swarm robotics. Terms like ‘swarm robotics’, ‘multi-agent systems’, and ‘swarm intelligence’ act as important pillars of the knowledge base, indicating the consolidation of the field that relies on the interaction between multi-agent systems and distributed robotics. The importance of concepts such as self-organization, stigmergy, and emergence testifies to the prevalence of complex system theory as the theoretical foundation. Simultaneously, recurrent occurrence of the bio-inspired metaheuristics such as ant colony optimization and particle swarm optimization, together with concepts such as algorithm, model, optimization, and learning, suggests the predominance of computational approaches related to collective coordination and decision-making. At the same time, concepts such as coordination, communication, environment, and distributed show the importance of local interactions between agents mediated by the environment. Moreover, concepts such as ant, collective, behavior, and dynamic indicate the significance of natural models used for the design of artificially created systems. These findings converge on three main axes—namely, collective robotic systems, computational methods, and bio-inspired approaches. The result of such an analysis is shown in Figure 4.

The relational structure that can be identified within the co-occurrence network allows understanding how the concepts relate to one another and how they are clustered by topic. Swarm robotics is at the heart of the application-oriented part of the network as the dominating theme; it demonstrates the strong interrelationship between swarm robotics and swarm intelligence, implying a biological transfer process to robotics. Additionally, a theoretical part of the cluster is represented by the concepts of multi-agent systems, self-organization, and stigmergy, suggesting that agent-based modeling forms a basis for researching distributed coordination. Terms like foraging, coordination, and ant colony optimization confirm the existence of classical problems in the field and show that social insect-based approaches continue to be relevant due to the combination of theoretical and applied aspects of research.

**Figure 5:**
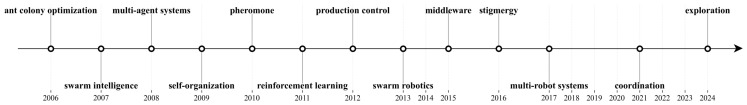
Timeline of the terms exhibiting the highest annual percentage growth, highlighting the evolution of the field from bio-inspired foundations toward advanced applications in distributed coordination and cooperative robotics.

The evolutionary dynamics of this area become even clearer in the context of burst-term analysis, where it is possible to trace the emergence of periods with rapid growth in scientific interest. Based on the data provided, the following path can be observed: bio-inspired meta-heuristics (e.g., ant colony optimization in 2006); establishment of swarm intelligence in 2007; an increase in the number of terms related to multi-agent systems, self-organization, and pheromones from 2008 to 2010; and methodological diversification in the form of reinforcement learning, production control, middleware, and swarm robotics during the 2010s. More recently, an increase in burst terms such as stigmergy, multi-robot systems, coordination, and exploration has occurred, as shown in Figure 5.

The examination of burst term publications offers insights into the effects of new concepts on the evolution of scientific literature within the sphere under study. With regard to the burst term ant colony optimization, the selected studies provide a number of valuable insights into the contribution of algorithms inspired by biology into solving tasks related to distributed planning and coordination. Specifically, Ref. [61] examined issues of efficient task allocation in robotic teams relying on the principle of local learning and adaptive choice of agents. Another milestone was represented by [121] who offered a novel approach towards global path optimization using HACO (hybrid ACO). In contrast to traditional approaches, HACO avoids premature convergence in the process of solving problems.

In connection with the burst term “swarm intelligence”, a number of interesting studies were found. Particularly, Ref. [35] provided a theoretical overview of biological mechanisms underlying social insects’ actions, while Refs. [77,79] were concerned with path optimization and bio-inspired communication mechanisms used by robots. In addition, the cited study by [35] achieved significant popularity in the sphere, having become one of the most cited publications. The term “multi-agent systems” implies a period of theoretical diversification and application of findings related to the autonomous and collective behavior of robotic agents. In particular, Ref. [111] demonstrated the possibility to apply an ant collective intelligence in order to solve the dynamic scheduling problem in industrial settings, while Refs. [67,122] investigated organizational aspects and autonomic services in relation to CAS. The term “self-organization” and other similar terms (“pheromone”) reflect a period of further consolidation of scientific knowledge and translation of ideas into practice. The studies such as [123,124,125] show examples of applications of theoretical principles related to stigmergy and regulation in multi-robot cooperation.

Finally, a number of burst terms, including “swarm robotics”, “multi-robot system”, “coordination”, and “exploration” imply that the focus has shifted to complex applications in dynamic and collective environments. As an example, Refs. [32,49] present research in the sphere of advanced navigation algorithms and collective robot control strategies, while Ref. [126] is devoted to the issue of human-robot interaction in the case of UAV swarms, focusing on scalability and autonomy aspects. Ref. [127] presents another important publication that emphasizes the role of distributed coordination and bio-inspired solutions to explore the environment. Clearly, the emergence of these terms indicates that they should not be seen as transient tendencies in research only. By contrast, these burst terms can be viewed as markers of major changes in the field of robotics.

## 7. Synthesis and Future Perspectives

The Synthesis and Future Perspectives section is dedicated to combining and analyzing the results obtained during this research to provide an integrated assessment of the existing body of knowledge about stigmergy and self-organizing systems in swarm robotics. This analysis includes the identification of central concepts discussed in the field, their relations, and the ways in which they have evolved over time. Thus, using the results of keywords, co-occurrence network, temporal trend, and burst term analysis, it becomes possible to identify the pattern through which the evolution of the science occurred, including well-established developments and directions that still need to be improved upon.

The importance of this analysis is rooted in the capacity to link findings from various dimensions in one coherent synthesis of the research landscape in stigmergy and self-organizing systems in swarm robotics. On the basis of recent literature, it is clear that research streams on collective intelligence, distributed coordination and control, and automation of multi-agent systems have been growing actively over the past five years. However, there are several areas where the science still lacks sufficient progress, such as scaling issues, robust operation in dynamically changing environments, and the combination of multiple approaches into hybrid solutions.

### 7.1. Main Contributions

First, impressive progress can be noted in collective coordination systems featuring multiple independent agents, where communications among dispersed entities are achieved through bio-inspired means like ant colony algorithms and swarm intelligence [17,18]. Several contributions related to UAVs confirm the efficiency of novel methodologies in dealing with problems such as search and cooperative navigation over unknown terrains, thus demonstrating the flexibility of solutions built around the application of local rules and distributed optimization [42,128]. Another important trend relates to the emergence of novel interfaces and concepts of collective autonomy allowing users to coordinate complex robot swarms effectively and at scale [129,130]. Overall, the mentioned contributions evidence methodological maturity, since their authors make use of advanced models combining control, perception, and visualization techniques.

Moreover, numerous studies have been dedicated to optimizing distributed systems, particularly in terms of dynamic task allocation in satellite systems and trust management in peer-to-peer structures [93,131,132]. The significance of the described works lies in highlighting the need for hybrid approaches based on bio-inspiration and adaptive heuristics, which improve their resilience to structural and environmental alterations. Such high citation counts also illustrate the contribution of these studies to the consolidation of the field, as far as both methods and theories are concerned. Concerning the issue of collective construction and exploration, contributions related to architectural robotics and 3D printing on the Moon show how robots can be used to solve sophisticated problems in these areas [133,134,135]. The relevance of swarm intelligence in the implementation of collective coverage, rules, and multi-objective optimization is clearly illustrated by the above contributions, as this shows how the field expands to tackle big and highly uncertain problems. Consequently, one should say that this literature review confirms that collective intelligence becomes an essential part of research in swarm robotics. Finally, distributed reinforcement learning techniques and autonomous trust-based cyber-physical systems are becoming increasingly popular, representing an interesting area for future research on real-time adaptive control in multi-agent systems [19,93]. Overall, the analysis of recent advancements proves their consistent character, since the field appears to have reached a considerable degree of scientific maturity in theory, methodology, and applications.

### 7.2. Open Challenges and Research Gaps

Although a series of relevant advances have been made in swarm robotics and self-organizing systems, many fundamental issues have prevented the maturation of this area. A central problem in self-organization is related to physical intelligence and computational intelligence. Although the literature has paid much attention to control and coordination algorithms, the study of the physical properties of the agents in order to optimize their behavior has been relatively scarce. This gap in the literature reveals the need for methods that could model and take advantage of the relationship between embodiment and control, thus enhancing the behavior of artificial systems to match those of biological entities [63].

In addition, a major limitation of the area is that of conceptual fragmentation and lack of experimental standardization. Several researchers use proprietary methodologies and assessment criteria, which hinder the comparability between different approaches. This issue is especially prevalent when studying bio-inspired or emergent strategies because there are considerable differences between experimental conditions, simulation parameters, and representation of the agents, yielding inconsistent findings and preventing the creation of a cohesive body of work [136]. Moreover, multi-scale modeling poses another critical challenge, since knowledge about cooperation and collective behaviors at multiple hierarchical scales is insufficient because of the difficulty involved in extrapolating experimental results obtained from controlled systems [136]. This limitation poses a challenge when creating algorithms for the robust coordination of heterogeneous robotic systems with significant environmental variability.

Another limiting factor of this literature is that of geographical and thematic concentration. Much of the research carried out has been performed in laboratory conditions or simulations, and little has been done regarding practical applications or large-scale studies, which constrains the generalizability of the results and could bias future investigations on whether certain strategies could be applied to real-world problems. It is therefore possible that relevant emergent phenomena will be overlooked by focusing on experimental conditions in which they do not occur. Last but not least, there are still relevant limitations regarding methodology, which is characterized by the absence of methodologies integrating physical modeling, collective intelligence, distributed optimization, and adaptive learning [63,136].

### 7.3. Future Research Directions

Based on the literature review, the development of swarm robotics is expected to be influenced by emerging trends associated with the study of collective intelligence, bio-inspired approaches, and integration into the cyber-physical system paradigm. It was noted that the combination of different distributed machine learning paradigms, optimization models based on swarm intelligence, and adaptive physical control techniques represents a very promising direction for improving the adaptivity and responsiveness of multi-agent robotic systems working in dynamic conditions [42,128]. The further development of relevant approaches and the creation of frameworks for managing multi-robot collaboration must be considered. The issues related to system scalability and robustness should not be overlooked either.

Medium-term prospects should be linked to interoperability among various robots with different capabilities. In particular, it may include cooperation between robots operating in the air, on the surface, and in outer space, as well as the integration of distributed sensor networks into the corresponding robotic ecosystem [93,131]. The synchronization of communication protocols and data processing tools will allow researchers to design efficient collaborative agent-based solutions to solve complex tasks related to environmental monitoring, rescue operations, or automated construction of objects in extreme conditions. At the same time, interdisciplinary expansion is one of the most important directions. Knowledge related to physics, biological processes, materials science, and even neuroscience will make it possible to use unique principles of adaptation and collective decision-making in order to ensure a higher efficiency in resource utilization, adaptation to environmental changes, and maintenance of collective behavior [63]. Consequently, there will be possibilities for designing self-organizing swarms suitable for cognitive manufacturing, distributed logistics, and ecosystem monitoring.

From a long-term perspective, many opportunities await in the realm of innovative optimization algorithms such as enhanced versions of Particle Swarm Optimization and Ant Colony Optimization applied to multi-objective, high-dimensional optimization and cooperative search problems [16,17]. The use of distributed reinforcement learning and physical feedback will increase the ability of systems to continuously learn and operate in changing environments. As a result, there will be more chances to prove that experimental results obtained using a certain algorithm can be generalized. In addition, from a long-term perspective, it is necessary to pay attention to methodological aspects, namely the development of unified standards, relevant benchmarks, and testing environments [20,103]. Such activities will contribute to the acceleration of scientific progress and help shape effective strategies for short, medium, and long-term research and policy development.

## 8. Conclusions

The current paper sought to consolidate and systematize the accumulated knowledge about stigmergy and self-organization mechanisms in the field of swarm robotics by analyzing key concepts, computational approaches, architectures, and applications. As a result, it can be concluded that, despite certain methodological and conceptual fragmentation, the discipline demonstrates clear structures that enable a comprehensive understanding of how the studied processes are formalized and utilized. The classification of the literature according to hierarchical levels reveals an epistemological evolution in which theoretical ideas contribute to the formation of the method and its practical application. It was found that the field has developed a well-consolidated framework built on the basis of such ideas as collective intelligence, stigmergy, and MAS, predominantly utilizing biologically inspired computational approaches. Moreover, a gradual evolution was identified within the discipline, going from purely conceptual ideas to the use of hybrid algorithms, distributed learning techniques, and swarm optimization, leading to the application of these mechanisms in UAVs, intelligent manufacturing, and other real-world environments. This pattern indicates not only the consolidation of indirect coordination and emergent processes as core aspects of the field but also its interdisciplinary nature and applicability to solving real-world problems.

Despite achieving considerable success and moving to an advanced stage of scientific maturity, many limitations still need to be addressed. These include insufficient standardization of experimental procedures, inadequate benchmarking efforts, difficulties in developing multi-scale models, and a lack of interaction between computational and physical intelligence, among others. Moreover, heterogeneous sensing capabilities, sensor noise, calibration issues, and limitations in perception range represent key challenges that affect the reproducibility and interoperability of swarm robotic systems. The uneven distribution of research production around the world and differences in the impact of studies might indicate certain gaps concerning global consolidation and widespread utilization of results. All these factors mean that significant challenges remain in achieving adequate comparability and reproducibility of research while transferring proposed solutions to practice. Thus, future research directions seem to be associated with such areas as the use of hybrid paradigms that incorporate reinforcement learning, swarm optimization, and adaptive control, the development of interoperable systems, benchmarking, and the creation of common standards. In addition, incorporating knowledge from other disciplines, such as physics, biology, and neuroscience, is likely to increase the adaptability and effectiveness of collective systems. Future research should also consider standardized approaches for sensing integration, environmental perception evaluation, and robust sensor design to ensure reliable stigmergic interactions and coordination in heterogeneous multi-robot systems. Consequently, this work contributes by synthesizing existing knowledge in the field and discussing its achievements, limitations, and potential research directions.

## Figures and Tables

**Table 1:** Comparison of the characteristics of related works. The columns represent: (A) Swarm Intelligence-based Systems, (B) Multi-Agent Systems, (C) Application of Metaheuristics, (D) Use of Artificial Intelligence or Reinforcement Learning, (E) Consideration of Technological Infrastructure, (F) Type of Application addressed in the studies. A check mark (✓) indicates that the corresponding characteristic is addressed in the study.

Reference	Topic	A	B	C	D	E	F
[16]	Target search using PSO	✓	–	✓	–	–	✓
[26]	Agent-based manufacturing systems	–	✓	–	✓	✓	✓
[17]	Evolution of PSO and ACO	✓	–	✓	–	–	✓
[24]	Cybersecurity in robots	–	✓	–	✓	✓	–
[21]	Communication in multi-robot systems	–	✓	–	–	✓	–
[22]	Swarm robotics concepts and platforms	✓	✓	–	–	✓	✓
[23]	UAV swarm trajectory planning	✓	✓	✓	–	✓	✓
[18]	Taxonomy of metaheuristics	–	–	✓	–	–	✓
[20]	UAV route planning	✓	✓	✓	✓	–	✓
[19]	Distributed learning in teams	–	✓	–	✓	–	✓
[27]	Cooperative multi-agent guidance	–	✓	–	–	✓	✓
[25]	UAV localization and sensing	✓	✓	–	✓	✓	✓
This review	Stigmergic and self-organized systems	✓	✓	✓	✓	✓	✓

**Table 2:** Scientific databases used for article collection. Searches were conducted between June 2025 and April 2026. All database URLs were first accessed on 1 June 2025.

Database	Advanced Search URL
IEEE Xplore	https://ieeexplore.ieee.org/search/advanced
ACM Digital Library	https://dl.acm.org/search/advanced
ScienceDirect	https://www.sciencedirect.com/search
Springer Nature	https://link.springer.com/advanced-search
MDPI	https://www.mdpi.com/search
Wiley Online Library	https://onlinelibrary.wiley.com/search/advanced

**Table 3:** Structure of the Boolean query used for article collection. The terms within each topic are combined using OR, and the three topics are combined using AND. The wildcard symbol (*) represents zero or more trailing characters, allowing the retrieval of different word variants.

Topic	Terms
Stigmergy	“stigmergy”
	“stigmergic”
	“indirect coordination”
	“environmental marking”
	“digital pheromone”
Self-Organizing	“self-organization”
	“self-organizing”
	“emergent behavior”
	“decentralized control”
	“collective behavior”
	“pattern formation”
Swarm Robotics	“swarm robotics”
	“swarm robot *”
	“multi-robot system *”
	“multi-agent system *”
	“robot swarm *”
	“collective robotics”

**Table 4:** Study selection according to the PRISMA 2020 process.

Database	Identified	Included
IEEE Xplore	3	0
ACM Digital Library	67	10
ScienceDirect	208	168
Springer Nature	138	119
MDPI	33	30
Wiley Online Library	21	11
Total	470	338

**Table 5:** Analytical axes adopted in the review and their corresponding study subsets.

Analytical Axis	Objective	Studies
Macrostructure of Scientific Production	Characterize publication patterns, geographical distribution, citation indicators, and collaboration networks.	338
Knowledge Base Analysis	Identify and organize the conceptual structure of the field through a hierarchical thematic taxonomy.	80
Topic Evolution and Emerging Trends	Detect emerging topics, conceptual bursts, and influential studies associated with temporal trends.	30
Synthesis and Future Perspectives	Identify current challenges, research gaps, and future research directions based on recent high-impact studies.	26

**Table 6:** Consolidated distribution of scientific production by country, providing an integrated view of publication volume, academic impact, and international collaborations. The columns represent, respectively, the total number of published articles (NA), total accumulated citations (NC), average citations per article (AC), median citations (MC), the highest number of citations recorded for a single article (MaxC), and the number of international collaborations (IC). The table highlights the concentration of scientific production, the heterogeneity of publication impact, and the centrality of specific countries as strategic hubs within the global research network. Boldface indicates the highest value in each quantitative column.

Country	NA	NC	AC	MC	MaxC	IC
Italy	**43**	1294	30.09	18	259 [31]	**15**
China	34	649	19.09	6	317 [32]	6
United States	29	**4461**	**153.83**	21	**2481** [33]	5
United Kingdom	24	1020	42.50	21	184 [34]	8
France	23	1169	50.83	16	467 [35]	10
Belgium	16	1750	109.38	44	764 [36]	10
Spain	13	1484	114.15	30	1033 [37]	0
Switzerland	11	653	59.36	**67**	146 [38]	9
Canada	11	548	49.82	18	345 [39]	9
Australia	11	549	49.91	15	260 [40]	0
India	13	810	62.31	24	369 [18]	0
Germany	10	835	83.50	36	378 [41]	3
UAE	6	159	26.50	10	86 [42]	0
Japan	6	77	12.83	11	27 [43]	0
Brazil	6	218	36.33	13	103 [44]	0

**Table 7:** Leading journals with the highest number of articles published in the analyzed corpus. The columns indicate, respectively, the journal name, the publishing organization, and the total number of published articles (NA).

Journal	Publisher	NA
Swarm Intelligence	Springer	18
Robotics and Autonomous Systems	ScienceDirect	16
Engineering Applications of Artificial Intelligence	ScienceDirect	12
Transactions on Autonomous and Adaptive Systems	ACM	9
Autonomous Agents and Multi-Agent Systems	Springer	8
Computers in Industry	ScienceDirect	8
Neurocomputing	ScienceDirect	7
Future Generation Computer Systems	ScienceDirect	7
Swarm and Evolutionary Computation	ScienceDirect	7
Artificial Life and Robotics	Springer	7
Applied Sciences	MDPI	7
Journal of Intelligent Manufacturing	Springer	6
Cognitive Systems Research	ScienceDirect	6
Sensors	MDPI	6
Autonomous Robots	Springer	6

## Data Availability

The dataset generated and analyzed during this study and the review protocol are publicly available in the GitHub repository https://github.com/LuigiRibeiro/SSOS-SR-Dataset (accessed on 1 June 2026). Analytic code will be made available upon reasonable request.
